# Exploring Hepatocellular Carcinoma Mortality Using Weighted Regression Estimation for the Cohort Effect in Taiwan from 1976 to 2015

**DOI:** 10.3390/ijerph19095573

**Published:** 2022-05-04

**Authors:** I-Shiang Tzeng, Jiann-Hwa Chen

**Affiliations:** 1 Department of Research, Taipei Tzu Chi Hospital, Buddhist Tzu Chi Medical Foundation, New Taipei City 23142, Taiwan; 2 Department of Statistics, National Taipei University, Taipei City 10478, Taiwan; 3 Department of Gastroenterology and Hepatology, Taipei Tzu Chi Hospital, Buddhist Tzu Chi Medical Foundation, New Taipei City 23142, Taiwan

**Keywords:** hepatocellular carcinoma, weighted cohort effect, regression model

## Abstract

To estimate the cohort effects that remove the efficacy of age and the period in the age-period statistics of a contingency table, the multiphase method is put forward. Hepatocellular carcinoma (HCC) is one of the most common malignancies of the liver. Understanding the predictive effects of age, period, and cohort on HCC mortality trends may help to estimate the future HCC burden, identify etiological factors, and advise public health prevention programs. Estimates of future HCC mortality and the associated health burden were forecast using an age–period–cohort (APC) model of analysis. By running a regression of residuals that were isolated from the median polish stage of cohort classification, the study controlled for HCC mortality confounding variables and interpreted time trends in HCC rates. The literature shows that the weighted mean estimation derived from the confidence interval (CI) is relatively restricted (compared to the equal-weighted evaluation). This study aimed to illustrate the effects of age, period, and cohort on the incidence and mortality rates, along with the weight equivalent to the segment of death number caused by HCC in each cohort. The objective of that work was to evaluate the proposed method for appraising cohort effects within the age-period data of contingency tables. The weighted mean estimate from the regression model was found to be robust and thus warrants consideration in forecasting future HCC mortality trends. The final phase was factored in to calculate the magnitude of cohort effects. In conclusion, owing to the relatively constricted CI and small degree of uncertainty, the weighted mean estimates can be used for projections based on simple linear extrapolation.

## 1. Introduction

Hepatocellular carcinoma (HCC) is regarded as a widespread and prime malignancy of the liver [1]. Worldwide, it ranks as the second greatest cause of mortality in men and sixth in women (14.7% and 6.5% of deaths for men and women, respectively) [2]. In Taiwan, it dominates the field of cancer, as it is the leading cause of cancer in men and the secondary cause of cancer in women (approximately 22% of men and 14% of women die of HCC) [3]. The estimates of the global incidence of HCC in 2000 were 398,000 men and 166,000 women per year [4].

The multiphase method for estimating cohort effects in age–period contingency table data, proposed by Keys and Li [5], takes three stages to conceptualize the cohort effects. Since the median polish does not lean on a specified allocation or formation, various types of mortality and trend data such as rates (of log transformation), proportions, and counts can be analyzed. The first stage is a graphical representation, which is followed by a median polish that is used to degrade log-additive components of ages or periods. A median polish analysis then makes up the second stage, whereby iteratively subtracting the median from each row and column detaches the additional effects of age and period. This is followed by linear regression of the residuals, factoring in cohort effects and random errors.

By intermittently subtracting the median from each row and column, the median polish may be used to delineate statistics in a two-way contingency table [6] and separate the supplementary influence from the age (i.e., row) and period (i.e., column). The first person who adopted APC analysis with the median polish [7] was Selvin. No specified allocation or structure of the data within a two-way contingency table means this technique can be used extensively for various types of data that consist of a table, such as suicide data [8]. Moreover, there is another advantage of the APC model, in that it can be applied to narrate the secular tendency in terms of disease incidence or mortality [9].

HCC has a close interrelationship with cirrhosis owing to alcohol consumption and viral etiologies [10]. It takes, on average, 20 years for cirrhosis to develop after the onset of hepatitis C virus infection [11]. Although malignant hepatocellular tumors do not usually metastasize, it is not easy to clear them when they do. In clinical practice, follow-up reviews are held at intervals of two to three months after the discovery of alpha-fetoprotein (AFP) by investigative criteria such as ultrasound, computed tomography, and magnetic resonance imaging. A novel biomarker used to detect HBV-related HCC is the HBV DNA quantitation-time index (HDQTI) [12]. The HDQTI is recognized as the optimal product in follow-up and logarithm, detecting the ratio of normal HBV DNA load. The novel HDQTI can be referred to as an independent prognostic indicator for recurrence in HBV-related HCC [13]. Most HCC patients present with a poor prognosis. It is thus critical to develop novel approaches, such as cell-based immune therapies [14]. These therapies are currently being evaluated in solid tumors including HCC, for example, chimeric antigen receptor T cells and T-cell receptor-engineered T cells.

Progression-free survival was demonstrated in a previous network meta-analysis, which studied the agents regorafenib, cabozantinib, and ramucirumab. Additionally, the outcomes of interest were a comprehensive benefit over the placebo, which lacked an advantage compared to any others [15]. Overall, for refractory patients, regorafenib and cabozantinib are the preferable options. Ramucirumab is deemed an ancillary option to treat [15] patients whose AFP levels are 400 ng/mL or higher. Accordingly, an amalgamation of immune agents integrating target drugs for the therapy of advanced HCC was endorsed by the Taiwan Food and Drug Administration on 1 August 2020.

Taking the time trend of HCC mortality into account, by simple linear extrapolation of the observational log in age-adjusted rates, an orthodox analysis may overlook some of the pivotal attributes that are hidden within the data (such as the cohort effects). During the period from 1976 to 2005 in Taiwan [16], if we conduct simple linear extrapolation of the long-term trends in HCC mortalities, there is no objective reason to doubt the trend, since it has been proliferating for 35 years and seems as though it will continue to escalate in the future. Yet, the current trend in HCC mortalities is tailing off. This can be attributed to the fact that the phenomenon is a consequence of the cohort effects that can be recognized in APC analysis.

In such context, this study aimed to explore the effects of age, period, and cohort on HCC mortality rates, along with the weight equivalent to the proportion of HCC death in each cohort [17]. In addition, we sought to identify weighted estimates for a cohort of the age–period data in contingency tables, which is advocated and should be considered as key to forecasting future HCC mortality trends.

## 2. Methods

### 2.1. Data Sources

To interpret the calculations, data on HCC mortality from 1976 to 2015 for both sexes were adopted to conduct the research in Taiwan from individual health records of the Ministry of Health and Welfare (MOHW). HCC mortality was categorized according to the International Classification of Disease (ICD) Code 150. Mortality data were accessible for three categories: ten five-year age groups (40–44, 45–49, 50–54, 55–59, 60–64, 65–69, 70–74, 75–79, 80–84, and 85+), eight five-year periods (1976–1980, 1981–1985, 1986–1990, 1991–1995, 1996–2000, 2001–2005, 2006–2010, and 2011–2015), and 17 birth cohorts (mid-cohort years: 1891, 1896, 1901, 1906, 1911, 1916, 1921, 1926, 1931, 1936, 1941, 1946, 1951, 1956, 1961, 1966, and 1971). Based on the above information, we computed the age-specific and age-adjusted mortality rates (using the 2000 World Standard Population) [18].

First, the mortality rate is denoted as *i*, age group as *j*, and the period group as *λ_ij_* in the following formula:

$$\begin{array}{c} \log\lambda_{ij}=\mu +\alpha_{i}+\beta_{j}+\gamma_{k}\;\;\;i=1,2,\ldots ,I,\\ j=1,2,\ldots ,J,\\ k=j-i+I, \end{array}$$

Within the APC model, the intercept term is denoted as *μ*, the age effects as *α_i_*, the period effects as *β_j_*, and the cohort effects as *γ_k_*.

$$\sum_{i}\alpha_{i}=\sum_{j}\beta j=\sum_{k}\gamma k=0$$

The subsequent constraints are thus availed.

### 2.2. Multiphase Method with Weighted Regression Model in Obtaining the Measure of Cohort Effects

The multiphase method follows a three-phase procedure, which allows for concrete evaluation of the cohort effect as a fragmentary interrelation of the data in the age–period contingency table [5,8]. Based on the log-additive effects as a constant term, with the addition of three elements—the age effect, period effect, and multiplicative interaction terms—we obtain the natural log rate (*λ_ij_*). As introduced in our previous research [19], there was a comprehensive description of the multiphase method with a weighted regression model in the context of gauging the cohort effects. The magnitude of cohort effects is calculated via the weighted regression of these residuals (*ε_k_*) within the cohort category in the final procedure (cohort is viewed as an indicator variable and is expressed as a collection of the m + n − 1 cohorts such as k = 1, 2, …, m + n − 1). Members belonging to the birth cohorts who were included are expressed as *W_k_*, symbolizing the weight of the *k*th cohort classification:

εk=Wk×γk+εijk

*ε_k_* is set up with the help of a vector from cohort effects (*γ_k_*) and error terms (*ε_ijk_*), where *ε_ijk_* stands for the error terms, and the unmeasured terms of age, period, and cohort categories are denoted as *i*, *j*, and *k*, respectively. The most extensively utilized factor in the number of deaths is the empirical weighting factor [20]. Furthermore, each of these weights is qualified for implementation in the regression equation. In line with the number of deaths caused by HCC in each cohort, the weighted average from the cohort effect can be demonstrated by the weight that is equivalent to the occupied proportion.

As stated in the mortality in the cohort in question, age was computed as the removed and unremoved influence of the cohort on deciding the reference categories. Moreover, in terms of cohort-specific mortality, there was minimal dissimilarity in the reference categories of cohorts with and without cohort influence. Following the previous research [5], after discarding influencing elements, the reference concerning the birth cohort could be ascertained based on the minute variation in its rate. It should be noted that we estimated the cohort effects for men and women.

## 3. Results

### 3.1. HCC Mortality Rates

**Figure 1:**
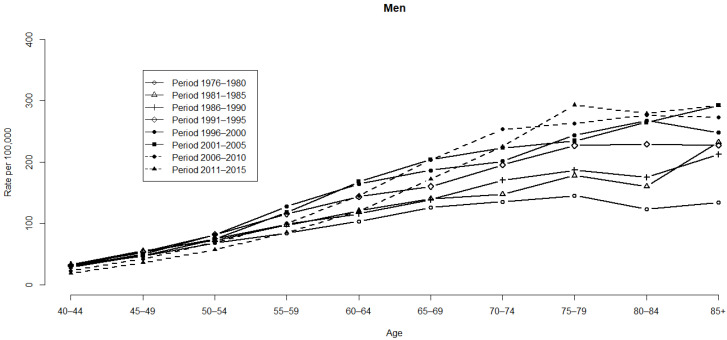
HCC mortality rates for age and period groups in men.

**Figure 2:**
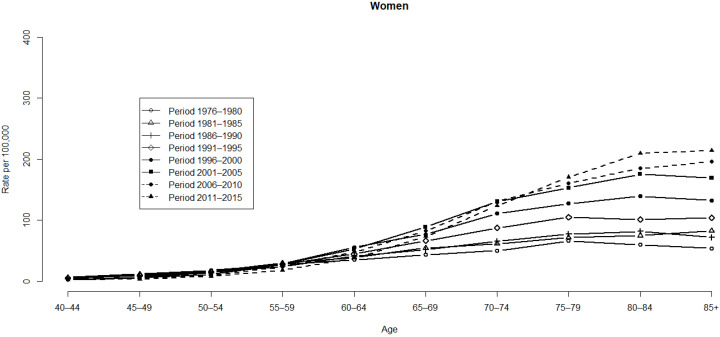
HCC mortality rates for age and period groups in women.

Figure 1 and Figure 2 illustrate the HCC mortality rates for age and period groups in men and women. Variations are more notable among men than women. HCC mortality rates begin in the 40–44 age group, as demonstrated by the distribution of rates based on age (refer to Figure 1). HCC mortality rates then increase consistently from the mid-age range of those aged ≥60 years (Figure 2). However, the HCC mortality rates (Table 1) based on age are substantially altered with time, which indicates a significant cohort effect concealed by the habitual age-period cross-classified vital statistics table. Even in the distant future, the cohort effect still cannot be discerned. As for the median polish procedure, we factored it into the logarithm transformation from the HCC mortality rates.

### 3.2. APC Model

The HCC mortality rates from the predicted cohort effects within the APC model are shown in Table 2 and Table 3, encompassing the weighted estimates obtained after following the weighted average procedure for both sexes. According to the smallest deviance (contrasted with the unweighted estimates), the range of the confidence interval (CI) was used as a criterion of accuracy. The unweighted effects of Table 2 provide the cohort effects under the birth cohorts. For men, the cohort effect rises from 0.73 (the earliest cohort effect in 1891) to 1.20 (the greatest cohort effect in 1936); for women (Table 3), the cohort effect rises from 0.68 (the earliest cohort effect in 1891) to 1.35 (the greatest cohort effect in 1936). Specifically, compared with the cohort in 1891, the cohort effects increase remarkably by approximately 51% and 68% for men and women, respectively. Conversely, the growth is evenly distributed in the weighted effects of Table 2, where the cohort effect increases from 0.71 (the earliest cohort effect in 1891) to 1.11 (the greatest cohort effect in 1936). In the same manner, the increased distribution for women is demonstrated in the weighted effects of Table 3, where the cohort effect rises from 0.64 (the earliest cohort effect in 1891) to 1.11 (the greatest cohort effect in 1926).

**Figure 3:**
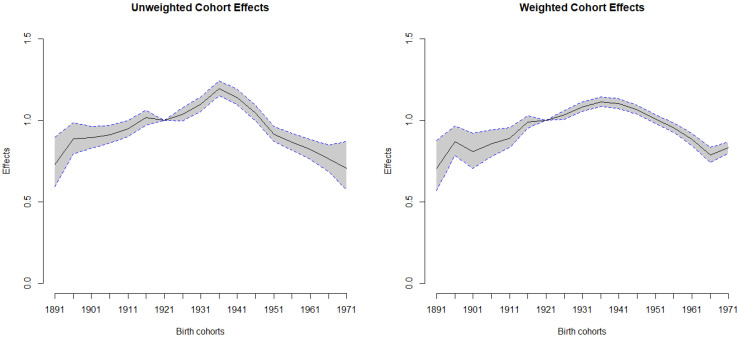
Weighted and unweighted cohort effects with 95% CI for men.

**Figure 4:**
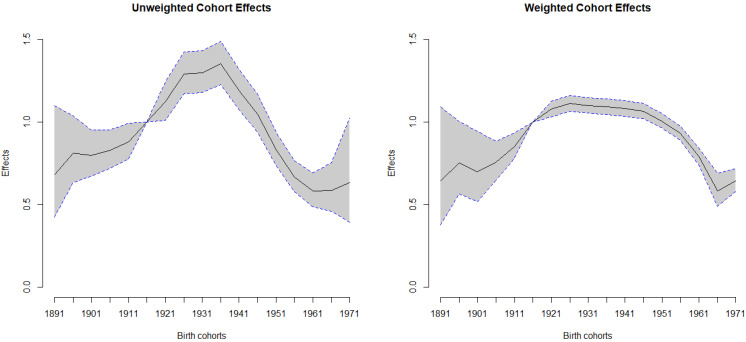
Weighted and unweighted cohort effects with 95% CI for women.

Among all birth cohorts, the individuals with the highest risk of HCC mortality were the men who were born in 1936 (Table 1). In weighted estimates, the effect was 1.11 (95% CI: 1.083–1.145) for the birth cohort in 1936, contrasted with the reference birth cohort in 1921. In the prior cohorts, a steeply declining trend was detected, along with the effects that were demonstrated after the cohort in 1936. Additionally, we plotted the weighted and unweighted cohort effects with 95% CI for men and women (Figure 3 and Figure 4). In both figures, it is clear that most of the widths within the 95% CI of the weighed cohort effects are shorter than those of the unweighted ones.

We placed a limit on our APC analysis within the median polish procedure to gauge the cohort effects, as well as the 95% CIs of the HCC mortality, and found that the residual errors (*ε_ijk_*) tended toward zero.

## 4. Discussion

**Figure 5:**
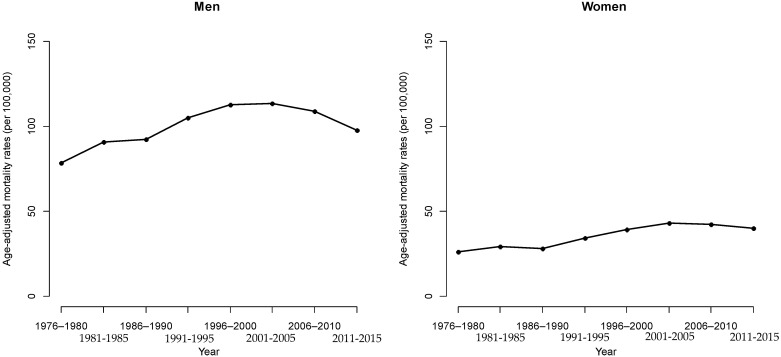
Long-term trends in HCC mortalities from 1976 to 2015 for men and women.

In summary, we found that most of the widths within the 95% CI of the weighed cohort effects were narrower than those of the unweighted ones. We also found the weighed cohort effects had a curvature trend that may dominate the long-term trends in HCC mortalities (Figure 5). In addition, this study may have identified significant cohort effects (or smaller standard errors) that should be considered in forecasting future HCC mortality trends.

We checked and compared our previously published [16] and newly updated extra 5-year data in this study to make some information about HCC trend. For males, the prediction of a previous publication [16] may overestimate the HCC mortality rate (refer to Figure 5). For females, the prediction of a previous publication [16] may underestimate the HCC mortality rate (refer to Figure 5). Furthermore, we found that the trend of the prediction of a previous publication [16] may coincide directly with a reverse trend by observation on HCC mortality. Moreover, we conducted a predicted pattern for the short-term period due to the addition of extra 5-year HCC data from a previous publication [19]. To simplify the predicted pattern, we focused on the linear-extrapolated period effects [16] for prediction of the next 5-year time period (i.e., 2016–2020). Age effects still were suited for all age groups of the next time period, 2016–2020. The prediction of cohort effects would extend to the 18th cohort effects but conservatively keep its value the same as the 17th estimated value (i.e., 1971 birth cohort estimated value). It can be found that the trend reverses itself at the period (i.e., 2016–2020) for men and women (Figure S1).

From the clinical perspective, as a consequence of the high morbidity, hepatitis B virus (HBV) infection is viewed as a vital health issue worldwide. There are approximately two billion people deemed contagious and 350 million people suffering from chronic HBV infection [21]. The most effective approach to prevent people from becoming infected is the hepatitis B vaccine. The earliest global hepatitis B large-scale vaccination program was put into practice in 1984 in Taiwan [22]. Pregnant women were screened using the HBsAg test, followed by HBeAg. Initially, the immunization scheme only included infants whose mothers were HBsAg carriers for the first two years. Every infant was then included in the third year of the vaccination program. Recently, the coverage ratio of hepatitis B vaccination increased to 99%. After receiving complete vaccine injections, approximately 90–95% of people will have acquired permanent immunity against the disease. Notably, owing to the effect of this worldwide vaccination program, there has been a decrease in pediatric HCC in Taiwan. Yet, the APC estimation still highlights cause for concern and caution regarding these growing trends, despite them having been reduced recently.

The trend of the cohort effect was investigated in this research by utilizing the median polish procedure. The weighted estimates permitted an estimation of the weighted average concerning the effect of the tapering CI in each cohort. Using the cohorts in 1936, these results were delineated in the form of cohort effects, since there are few modifications in HCC mortality rates that are not influenced by the cohort, as shown in these categories.

Amid the majority of modeling methodologies (e.g., models for linear or nonlinear regression), one of the ordinary presuppositions is that every authentic value in the data provides an estimation of the parameters inside an assured model with identical information. Accordingly, this signifies that the standard deviation from the error term is also known as the constant predictor variable. As investigated in our literature review, the assumption does not impose limits on modeling to estimate the parameters empirically. A smaller number of weights are supplied with slightly imprecise data points, and a greater number of weights are supplied with precise data points. In addition, being able to limit the standard deviation of the estimator is viewed as a benefit of the weighted procedure. Nonetheless, drawbacks of the weighted regression approach are often seen in empirical practice. In addition, the estimation of weight cannot be deployed to gauge the parameters, given that the correct weight figure is rarely known. Previous experience indicates that as a result of the estimation, the weighting is barely alterable and often is unaffected in the analysis or interpretation of regression [23].

Theoretically, when it comes to the APC model, diseases with rates dominated by age, period, and cohort effects comply. Furthermore, weighted-average estimates can be implemented for prediction [12,24,25]. It is generally recognized that the narrower the CI, the smaller the uncertainty.

In our study, there were numerous limitations. First, we could only surmise the etiologies of the changes being observed during the research. According to the age, period, and cohort effects, HCC mortality is amenable to the APC model. Nevertheless, in this work, the presence of immutable suppositions for the median polish that we used should be taken into consideration. Second, APC analysis can be applied extensively in the field of epidemiology for populations living in developing or developed countries but has restrictions for long-running cohort studies. Third, for confounders such as comorbidity and lifestyle inside the APC model, the information from the aggregate dataset was not adequate for us to modify; additional studies using individual data are essential to resolve these restrictions. Fourth, when applying the regression procedure under the multiphase approach, the number of deaths attributed to HCC was utilized as the weight. Applying diverse weights may spark minor inflation of estimated cohort effects, as the explicit weight is almost pending. Forth, caution should be taken when comparing the HCC mortality trend between men and women given that the reference years are not the same among genders. Lastly, this type of circumstance may be compounded while adopting differing estimations of APC methods to tackle non-identifiable factors (e.g., Holford uses the linear and curvature trends to study a non-identifiable problem [26]). The median polish makes intricate conjectures concerning APC models by computing the cohort effect with minimal assumptions, simply adopting a typical format for contingency tables.

In conclusion, in terms of adopting the regression model, the weighted estimate permits the effect of a weighted average with a narrower CI to be studied in every cohort. Overall, using the weighted estimate in the regression model is recommended in practice. This will support future research into health policy and preventative health strategies in Taiwan.

## Figures and Tables

**Table 1:** Age–period contingency table of HCC mortality rate per 100,000 among men and women, Taiwan, 1976–2015.

		1976–1980	1981–1985	1986–1990	1991–1995	1996–2000	2001–2005	2006–2010	2006–2015
Men	40–44	31.41	33.10	33.20	31.24	30.40	29.10	23.26	19.30
	45–49	46.50	55.13	52.88	53.61	49.79	47.66	42.25	36.25
	50–54	68.11	72.47	74.56	81.73	81.65	74.19	69.12	56.96
	55–59	84.12	97.32	98.96	115.57	127.55	117.70	100.19	85.59
	60–64	103.58	120.45	115.71	143.74	164.14	168.20	145.35	119.83
	65–69	126.15	140.12	138.76	160.29	186.58	204.31	203.50	172.15
	70–74	135.44	147.79	170.44	195.56	201.40	223.00	253.46	225.23
	75–79	145.41	178.22	186.70	226.86	243.87	234.63	263.24	293.21
	80–84	123.63	160.33	175.09	229.23	267.55	264.61	276.39	278.95
	85+	133.97	232.56	212.33	227.57	248.07	292.51	272.51	291.86
Women	40–44	6.75	6.14	4.90	5.11	3.46	2.88	3.03	2.44
	45–49	11.99	11.10	9.21	9.76	6.49	5.76	4.90	3.79
	50–54	18.20	16.23	14.78	15.53	14.15	11.78	10.20	8.17
	55–59	27.57	30.06	24.45	28.43	28.65	28.04	23.68	18.34
	60–64	35.23	39.48	41.09	44.75	55.75	52.97	48.01	37.94
	65–69	43.49	54.68	52.09	66.36	76.92	89.05	82.80	73.44
	70–74	50.40	61.49	65.48	87.47	111.46	130.60	131.32	123.96
	75–79	66.47	72.02	77.57	105.09	127.37	153.26	160.98	170.88
	80–84	60.12	74.79	81.83	101.26	139.41	175.41	184.90	209.80
	85+	54.22	82.91	71.93	103.98	132.42	169.12	196.03	214.09

**Table 2:** Estimated rate ratios and 95% conference intervals for effect of birth cohort on hepatocellular carcinoma mortality of men in Taiwan, 1891–1971.

Cohort	Unweighted		Weighted	
(1891~1971)	Effects	95% CI for Effects	Effects	95% CI for Effects
1891	0.73	0.591–0.898	0.71	0.567–0.877
1896	0.88	0.795–0.986	0.87	0.784–0.967
1901	0.89	0.828–0.962	0.81	0.706–0.922
1906	0.91	0.859–0.967	0.85	0.777–0.940
1911	0.95	0.901–0.998	0.89	0.832–0.956
1916	1.01	0.970–1.062	0.99	0.950–1.027
1921	1.00	REF	1.00	REF
1926	1.04	0.997–1.079	1.03	1.007–1.060
1931	1.10	1.055–1.142	1.08	1.056–1.111
1936	1.20	1.149–1.243	1.11	1.083–1.145
1941	1.14	1.094–1.190	1.10	1.072–1.131
1946	1.04	0.997–1.092	1.06	1.036–1.093
1951	0.91	0.868–0.961	1.00	0.978–1.033
1956	0.87	0.817–0.921	0.96	0.927–0.985
1961	0.82	0.761–0.884	0.88	0.847–0.922
1966	0.76	0.685–0.849	0.79	0.740–0.834
1971	0.71	0.573–0.870	0.83	0.796–0.866

Note: REF = reference; CI = confidence interval.

**Table 3:** Estimated rate ratios and 95% conference intervals for effect of birth cohort on hepatocellular carcinoma mortality of women in Taiwan, 1891–1971.

Cohort	Unweighted		Weighted	
(1891~1971)	Effects	95% CI for Effects	Effects	95% CI for Effects
1891	0.68	0.423–1.099	0.64	0.378–1.090
1896	0.81	0.632–1.038	0.75	0.564–1.001
1901	0.80	0.669–0.951	0.70	0.516–0.944
1906	0.83	0.718–0.953	0.76	0.648–0.884
1911	0.88	0.778–0.994	0.85	0.779–0.935
1916	1.00	REF	1.00	REF
1921	1.12	1.011–1.243	1.08	1.031–1.125
1926	1.29	1.169–1.422	1.11	1.065–1.159
1931	1.30	1.178–1.432	1.10	1.053–1.147
1936	1.35	1.224–1.490	1.09	1.044–1.139
1941	1.19	1.073–1.320	1.08	1.035–1.129
1946	1.05	0.937–1.170	1.06	1.019–1.112
1951	0.83	0.735–0.939	1.00	0.961–1.049
1956	0.67	0.579–0.768	0.93	0.890–0.977
1961	0.58	0.487–0.692	0.79	0.740–0.843
1966	0.59	0.458–0.752	0.58	0.490–0.689
1971	0.63	0.394–1.023	0.64	0.577–0.721

Note: REF = reference; CI = confidence interval.

## Data Availability

The data that support the findings of this study are available in Table 1 of this article.

## Supplementary Materials

The following supporting information can be downloaded at: https://www.mdpi.com/article/10.3390/ijerph19095573/s1, Figure S1: Observation and prediction of age-adjusted mortality in HCC for men and women in Taiwan.
