# Supplementary material for: Exploring Hepatocellular Carcinoma Mortality Using Weighted Regression Estimation for the Cohort Effect in Taiwan from 1976 to 2015
Source: Int J Environ Res Public Health. 2022 May 4;19(9):5573. doi: 10.3390/ijerph19095573 (PMC9099783; doi:10.3390/ijerph19095573)
Supplement: Supplementary file 1 [file ijerph-19-05573-s001.zip › ijerph-1586542-supplementary.pdf]

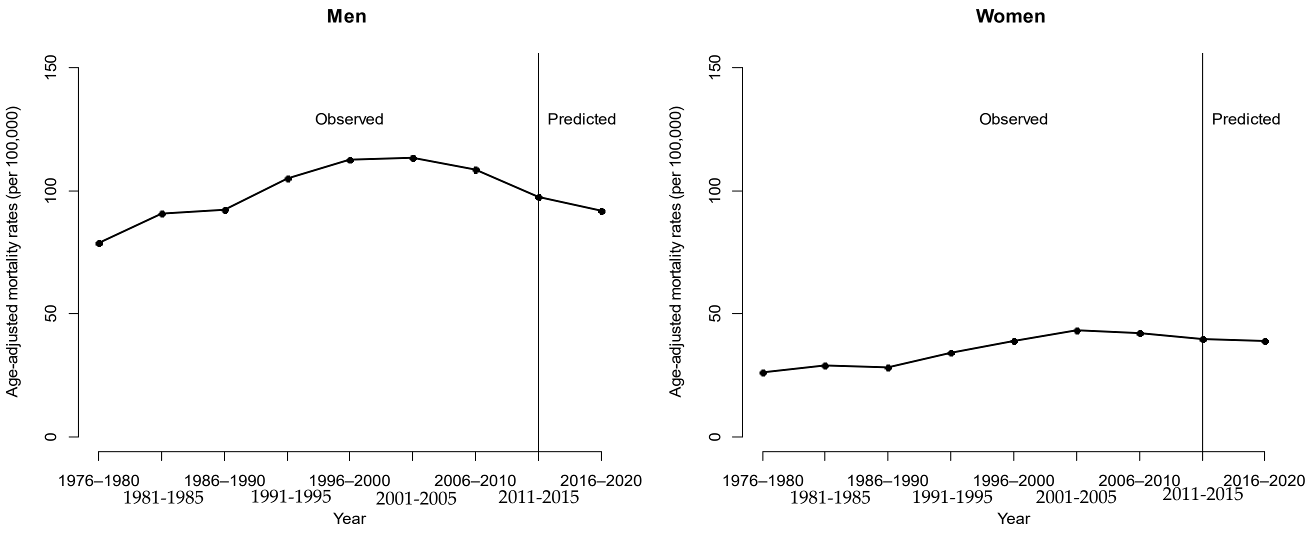

Figure S1. Observation and prediction of age-adjusted mortality in HCC for men and women in Taiwan.
